# The genetic and genomic background of multiple myeloma patients achieving complete response after induction therapy with bortezomib, thalidomide and dexamethasone (VTD)

**DOI:** 10.18632/oncotarget.5718

**Published:** 2015-11-09

**Authors:** Carolina Terragna, Daniel Remondini, Marina Martello, Elena Zamagni, Lucia Pantani, Francesca Patriarca, Annalisa Pezzi, Giuseppe Levi, Massimo Offidani, Ilaria Proserpio, Giovanni De Sabbata, Paola Tacchetti, Clotilde Cangialosi, Fabrizio Ciambelli, Clara Virginia Viganò, Flores Angela Dico, Barbara Santacroce, Enrica Borsi, Annamaria Brioli, Giulia Marzocchi, Gastone Castellani, Giovanni Martinelli, Antonio Palumbo, Michele Cavo

**Affiliations:** ^1^ ”Seràgnoli” Institute of Hematology, Department of Experimental, Diagnostic and Specialty Medicine (DIMES), Bologna University School of Medicine, Bologna, Italy; ^2^ Department of Physics and Astronomy (DIFA), Bologna University, Bologna, Italy; ^3^ Clinica Ematologica, DISM, University of Udine, Udine, Italy; ^4^ Clinica di Ematologia, A.O.U. Ospedali Riuniti di Ancona, Ancona, Italy; ^5^ U.O Oncologia Medica, Ospedale di Circolo e Fondazione Macchi, Varese, Italy; ^6^ Ematologia Clinica, A.O.U. Ospedali Riuniti, Trieste, Italy; ^7^ Hematology Division UTMO, Azienda “Ospedali Riuniti Villa Sofia-Cervello” Presidio Ospedaliero V.Cervello, Palermo, Italy; ^8^ S.C.Oncologia Medica, A.O. Sant'Antonio Abate, Gallarate, Varese, Italy; ^9^ Unità Operativa di Ematologia, Istituti Ospitalieri di Cremona, Cremona, Italy; ^10^ Myeloma Unit, Division of Hematology, University of Torino, A.O.U. Città della Salute e della Scienza di Torino, Torino, Italy

**Keywords:** multiple myeloma, gene expression profile, SNPs, VTD, complete response

## Abstract

The prime focus of the current therapeutic strategy for Multiple Myeloma (MM) is to obtain an early and deep tumour burden reduction, up to the level of complete response (CR). To date, no description of the characteristics of the plasma cells (PC) prone to achieve CR has been reported. This study aimed at the molecular characterization of PC obtained at baseline from MM patients in CR after bortezomib-thalidomide-dexamethasone (VTD) first line therapy.

One hundred and eighteen MM primary tumours obtained from homogeneously treated patients were profiled both for gene expression and for single nucleotide polymorphism genotype. Genomic results were used to obtain a predictor of sensitivity to VTD induction therapy, as well as to describe both the transcription and the genomic profile of PC derived from MM with subsequent optimal response to primary induction therapy.

By analysing the gene profiles of CR patients, we identified a 5-gene signature predicting CR with an overall median accuracy of 75% (range: 72%–85%). In addition, we highlighted the differential expression of a series of genes, whose deregulation might explain patients' sensitivity to VTD therapy. We also showed that a small copy number loss, covering 606Kb on chromosome 1p22.1 was the most significantly associated with CR patients.

## INTRODUCTION

Multiple Myeloma (MM) is a B-cell malignancy characterized by the proliferation in the bone marrow of clonal plasma cells, which acquire resistance to therapy as a result of their interaction with the bone marrow microenvironment. The natural history of MM is usually marked by multiple phases of relapse, which ultimately lead refractory patients to death. The aim of current myeloma therapy is to deepen the magnitude of response and to delay or prevent relapses [1, 2]. For this purpose, induction, consolidation and/or maintenance therapy are integral components of the modern treatment paradigm for MM patients [3–6]. The prime focus of induction therapy is to reduce the tumour burden as much and as early as possible, shrinking the residual disease to the level of complete response (CR) [7]. Recent incorporation of first and second generation immunomodulatory derivatives and proteasome inhibitors into newer induction regimens has enhanced the rate of CR compared to previous conventional chemotherapy, thus leading to a substantial improvement of progression-free survival (PFS) and overall survival (OS) [8–13]. Achievement of high-quality response to induction therapy given before autologous stem cell transplantation (ASCT) is an early and independent prognosticator of favourable post ASCT outcomes and represents a primary goal of current treatment strategies [14, 15].

However, despite evolution of modern therapeutic approaches, the prognosis of MM still remains variable, mostly driven by genetic abnormalities of the tumor clone [16, 17] and a proportion of patients fail to benefit from novel agents [18, 19]. Although identification of these patients might be relevant for selecting *a priori* the best treatment strategy, few biomarkers of response to novel agent-based therapies are currently available [20, 21].

The GIMEMA MMY-3006 study comparing Bortezomib-Thalidomide-Dexamethasone (VTD) with Thalidomide-Dexamethasone (TD) as induction therapy before, and consolidation after, double ASCT provided demonstration of the superior rate of response and PFS offered by the triplet VTD combination [8]. In particular, on an intention-to-treat basis, the rate of CR and near CR after induction therapy, the primary study end point, was approximately 3 times higher with VTD compared to TD. Based on these results, we performed a biological sub-study aimed at identifying the transcription profile and genomic background of patients who either achieved or failed CR after VTD induction therapy.

For this purpose, primary tumour cells obtained at diagnosis from these patients were globally profiled for both gene expression and single nucleotide polymorphism (SNP) genotypes, using an Affymetrix platform (Affymetrix, Santa Clara, CA). By evaluating gene expression profiling, we obtained a 5-gene signature, which predicted the achievement of CR to VTD induction therapy. We then succeeded in identifying both a transcription and a genomic profile distinguishing – right from diagnosis - patients with MM who will respond optimally to primary induction therapy with VTD. Annotation analysis was applied to reveal which biological functions critically influenced clinical response.

## RESULTS

### Response to induction therapy

For patients enrolled in the GIMEMA-MMY-3006 clinical trial, the achievement of CR after induction therapy was an early and independent predictor for prolonged PFS after ASCT. (median: 81 vs. 45 months, *p* = 0.0011) and independently affected outcomes in a multivariate analysis along with advanced ISS stage, presence of t(4;14) and/or del(17p) and arm of treatment.

Among patients receiving VTD induction therapy, CR was achieved in 15 patients (12.7%), while in the remaining 103 patients the following response categories were identified: near CR (nCR, 14 patients, 11.8%), Very Good Partial Response (VGPR, 40 patients, 33.9%), Partial Response (PR, 42 patients, 35.6%) and Stable Disease (SD, 7 patients, 5.9%). The most relevant clinical characteristics at baseline were comparable among patients who achieved CR and those who failed this objective, including t(4;14) (p14;q32) and/or del(17p) frequency, as evaluated by FISH, which was higher, although not at a statistically significant level, in CR compared to <CR patients (36% vs. 22.5%).

### Development of a GEP predictor for attainment of CR after VTD induction therapy

The main objective of the study was to obtain a gene-based classifier to be used to predict CR to VTD induction therapy. For this purpose, we compared the transcription profiles of patients who achieved CR vs. those of patients who failed this objective and results were analysed, in order to get an optimal-performing low-dimensional signature for classifying the response to therapy.

The analysis resulted in identification of several predictive signatures, among which a 5-gene one (Table 1) performed best in predicting CR, with an overall median accuracy of 75% (range: 72%–85%). The signature median sensitivity was 87% (range: 66%–87%), the median specificity was 73% (range: 73%–84%), the median Positive Predictive Value (PPV) was 31% (range: 28%–44%) and the median Negative Predictive Value (NPV) was 97% (range: 97%–98%). Of note, the profiled samples had been collected before any therapy, thus emphasising the predictive competence of the signature in identifying the response to therapy. Genes and probes included in the signature are detailed in Table 1: all but one probe were differentially expressed in CR patients (*p* < 0.05), albeit with minimal fold change (FC) fluctuations, thus suggesting that the classification power depends only on the multidimensional signature features as a whole, rather than on single gene classification power.

**Table 1:** Genes included in the 5-gene signature

Gene	Affy ID	FC	*p*	Description	Cytoband
***ACTR2***	200728_at	0.6	0.002	ARP2 actin-related protein2 homolog	2p14
***BAI2***	204966_at	−0.2	0.0006	brain-specific angiogenic inhibitor 2	1p35
***ANK3***	228766_at	−0.5	0.07	ankyrin 3, node de Ranvier	10p21
***GALNT5***	229555_at	−0.2	0.01	polypeptide N-acetylgalactosaminyl transferase 5	2p24.1
***GLT1D1***	229770_at	−0.2	0.007	glycosyl transferase 1 domani containing 1	12q24.33

In order to validate the results, the 5-gene predictive signature was tested on two independent, previously published, datasets: the first one (GSE55145) was generated using the Affymetrix Exon 1.0 ST platform from newly diagnosed MM patients enrolled in the IFM II clinical trial [22] and included 67 patients, 10 of whom (15%) obtained CR after bortezomib-dexamethason induction therapy. The second one (GSE9872) was generated using the Affymetrix HG-U133 A/B platform from relapsed MM patients enrolled in the APEX/SUMMIT clinical trials [23] and included 181 patients, 12 of whom (7%) obtained CR after salvage therapy with bortezomib as a single agent. Even though several discrepancies exist between the three compared datasets (mainly regarding disease stage, therapy and microarray platforms), the 5-gene signature median prediction accuracies were - in the best case - 79% in the IFM II dataset and 72% in the APEX/SUMMIT one (see Table 2 for performance details).

**Table 2:** Summary of the 5-gene signature's performances, as evaluated in two previously published dataset

Data set	Trial	Pts (N, disease phase)	Therapy	RR	Accuracy (median, range)	Sensitivity (median, range)	Specificity (median, range)
**GSE55145**	IFM II	67 (newly diagnosed)	VD	15%	66.7 (49.1–78.9)	60.0 (10.0–80.0)	65.7 (50.7–76.1)
**GSE9872**	APEX/SUMMIT	181 (relapsed)	V	7%	59.8 (47.9–71.0)	38.5 (8.0–84.6)	58.2 (46.7–68)

pts = patients; N = number; VD = velcade/dexamethasone; V = velcade; RR

### Transcriptional program of patients who achieved CR after VTD induction therapy

The transcription profile of patients who achieved CR was further analysed, in order to identify the biological processes most significantly conditioning the CD138+ phenotype of patients achieving CR with the triplet VTD combination. Several comparisons among gene profiles of patients stratified according to the depth of response were set up, with purpose view to identifying the most informative transcription profile describing the genetic environment of patients who achieved CR after induction therapy. These analyses finally resulted in identification of 5172 probes, representing 4281 genes according to the Affymetrix annotation database, significantly diverging in their expression among the most opposite response classes, i.e. CR (15/118 samples) and PR/SD (49/118 samples) (*p* < 0.05). Indeed we assumed that the clinical-based definition of these opposite response classes might actually reflect a peculiar biological feature of the plasma cell, as well.

In order to better understand the data in the context of MM, the list of differentially expressed genes was analysed with the GeneGo^®^ software filtering tool, to enrich for genes already known to be related to hematological diseases, thus obtaining a final list of 539 differentially expressed genes, with a fold change (FC) ranging from −2.656 to 3.303 (Supplementary Table S1). Of these, 363 genes were down-regulated, whereas 176 were over-expressed in patients achieving CR after VTD. Genes did not cluster on a specific chromosome, though chromosomes 1, 2, 19 and 17 (carrying 9.9%, 7.3%, 7.3% and 6.5% of the 539 differentially expressed genes, respectively) were particularly over-represented. By contrast, less than 2% of genes were located on chromosomes 15, 21 and 13.

Table 3 summarizes several of the top genes most significantly deregulated in CR patients, as compared to patients with PR/SD. Of interest, *CCND2* and *CCND1* expression proved to be mutually exclusive in CR patients, where they showed the highest and the lowest FC, respectively. Overall, MM plasma cell exhaustion, which characterizes patients who responded to VTD achieving CR, might be biologically accounted for by the deregulation of MM's key biology pathways, i.e. NFkB, (a), IL6R, VEGF and IGF1R signalling pathways; (b) basic cell processes (cell cycle and apoptosis regulation, cell homing and migration); (c) other signal transduction pathways related to initiation of RNA translation (i.e. eiF2, eiF4 and p70S6K signalling), to DNA damage control (p53 signalling) and to inflammatory response (CD40 signalling).

**Table 3:** Top-5 down- and top-5 over-expressed genes in patients who achieved CR, as compared to patients who achieved PR/SD

Gene	Affy ID	FC	*p*	Description	Cytoband
***CCND1***	208712_at	−2.217	0.0189	G1/S-specific cyclin-D1	11q13
***DKK1***	204602_at	−1.868	0.0345	Dickkopf-related protein 1	10q11.2
***JAK1***	1552611_a_at	−1.245	0.0291	Janus kinase 1	1p32.3-p31.3
***RUNX3***	204198_s_at	−0.958	0.0399	runt-related transcription factor 3	1p36
***TGFB1***	203085_s_at	−0.903	0.000495	transforming growth factor beta 1	19q13.1
***IKZF1***	205039_s_at	−0.802	0.0105	IKAROS family zinc finger 1 (ikaros)	7p13-p11.1
***CCND2***	200953_s_at	3.307	0.000612	G1/S-specific cyclin-D2	12p13
***ITGB1***	1553678_a_at	1.117	0.0301	integrin beta-1	10p11.2
***ENTPD1***	207691_x_at	1.068	0.0409	CD39 antigen	10q24
***CAV1***	212097_at	0.867	0.0219	caveolin-1	7q31.1
***HELLS***	223556_at	0.841	0.0138	proliferation-associated SNF2-like protein	10q24.2
***YES1***	202932_at	0.805	0.0354	Yamaguchi sarcoma viral oncogene homolog 1	18p11.31-p11.21

### Genomic profile of patients who achieved CR after VTD induction therapy

To complete the high throughput description of the baseline background of patients who achieved CR after VTD induction therapy, we finally performed a whole CNA profile by SNPs array in 89 patients for whom enough DNA was available.

Overall, the CNA profile confirmed the typical MM plasma cell distribution of numerical abnormalities [24–26], where a gain in odd-numbered chromosomes - characteristic of so-called hyperdiploid myeloma - was one of the most frequent CNAs observed (43 samples, 49%), along with *Rb1* CN loss on chr.13q14.2 (detected in 48 patients, 53,9%), *CKS1B* CN gain on chr.1q32.1 (detected in 28 patients, 31,4%), *CDKN2C* CN loss on chr.1p32.3 (detected in 10 patients, 11.2%), and *TP53* CN loss on chr.17p13.1 (detected in 9 patients, 10,1%).

**Figure 1:**
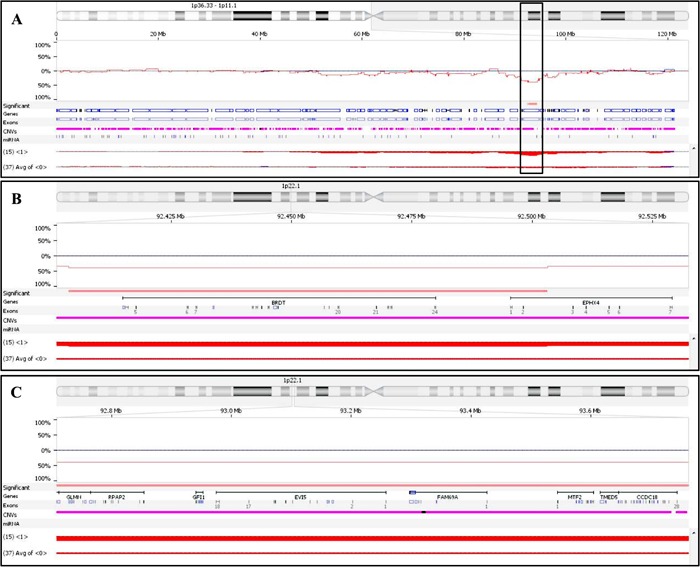
Comparison of chromosome 1 CNVs present in CR and PR/SD patients **A.** detailed view of the chromosome 1 short arm; the box highlights the two close chromosomal regions more frequently deleted in CR patients, as compared to PR/SD ones (*p* = 0.006). **B.** detailed view of the most telomeric small deleted region (99.5 Kb), covering *BRDT* and part of *EPHX4*. **C.** detailed view of the most centromeric deleted region (1,677.3 Kb), covering 20 genes, including *EVI5*.

In order to focus on the genomic background of patients who achieved CR and to identify chromosomal lesions potentially influencing response to induction therapy, we then used Nexus Copy Number software to compare the CNA profile of patients in CR and PR/SD (15/89 vs. 37/89 patients): 111 CNAs (41 CN gains, 24 CN losses and 46 LOH events) proved significantly associated with VTD maximal sensitivity (*p* < 0.05, 25% differential threshold). Of these, only 21 lasted when more stringent comparison criteria were applied (*p*-value < 0.01 and 10% differential threshold); an overall description of the 21 CNAs, including genes affected by each chromosomal aberration, is shown in Table 4. Two small CN losses on chromosome 1p22.1, covering 99 and 1,677 Kb, respectively, and carrying 2 and 20 genes, respectively, were the most significantly associated with CR patients, both being present in 60% of CR and in 19% of PR/SD patients, respectively (*p* = 0.006) (Figure 1).

**Table 4:** CN events characterizing patients achieving CR as compared to patients achieving ≤PR; for each CN lesion, the table lists the cytoband location, the dimension, the frequency in both subgroups of patients, the p level of significance and the genes included

Event	Cytoband Location	Region Length (bp)	Freq. in <1> (%)	Freq. in Avg of <0>(%)	*p*-value	Genes included within the CNA
**CN gain**	chr1q31.3	911	66,667	19,444	0,0025	*CFH*
	chr3q26.32	170	46,667	8,333	0,0039	*PIK3CA*
	chr1q31.3	1273	66,667	22,222	0,0040	*CFH*
	chr2p16.1	82365	26,667	0,000	0,0055	*CCDC85A*
	chr3q13.33	49065	46,667	11,111	0,0090	*PLA1A, POPDC2*
	chr3q21.2	413	46,667	11,111	0,0090	*KALRN*
	chr3q26.32	93	46,667	11,111	0,0090	*PIK3CA*
	chr3q26.32	1318	46,667	11,111	0,0090	*PIK3CA*
**CN loss**	chr1p22.1	25360	53,333	11,111	0,0026	*TGFBR3*
	chr1p22.1	606987	60,000	16,667	0,0052	BRDT, EPHX4, SETSIP, BTBD8, KIAA1107, C1orf146, GLMN*, RPAP2, GFI1*, EVI5*
	chr4p16.3	1421	26,667	0,000	0,0055	ZNF595, ZNF718*
	chr8p23.2	5038	26,667	0,000	0,0055	*CSMD1*
	chr8p22	502452	33,333	2,778	0,0063	*SGCZ*
	chr8p22	206245	33,333	2,778	0,0063	*SGCZ*
	chr8p23.1	665562	33,333	2,778	0,0063	*SGK223, CLDN23, MFHAS1*
	chr8p23.1	1049007	33,333	2,778	0,0063	ERI1, MIR4660, PPP1R3B*, LOC157273, TNKS, MIR597, LINC00599, MIR124–1, MSRA
	chr8p23.1 - p22	1028933	33,333	2,778	0,0063	*MIR3926–1, MIR3926–2, LONRF1, LOC340357, LINC00681, KIAA1456, DLC1, C8orf48*
	chr1p22.1	1372892	60,000	19,444	0,0077	EVI5*, RPL5, SNORD21, SNORA66, FAM69A, MTF2*, TMED5*, CCDC18*, LOC100131564, DR1*, FNBP1L, LOC100129046, BCAR3*, MIR760, DNTTIP2, GCLM*
**LOH**	chr4p12	225913	40,000	5,556	0,0053	*GABRG1*
	chr2q24.1	720756	26,667	0,000	0,0055	*GALNT5, ERMN, CYTIP*
	chr3q11.2	596327	26,667	0,000	0,0055	*LINC00879*
	chr4p16.3	1244341	26,667	0,000	0,0055	*ZNF595, ZNF718, ZNF876P, ZNF732, ZNF141, ABCA11P, ZNF721, PIGG, PDE6B, ATP5I, MYL5, MFSD7, PCGF3, LOC100129917, CPLX1, GAK, TMEM175, DGKQ, SLC26A1, IDUA, FGFRL1, RNF212, TMED11P, SPON2, LOC100130872, CTBP1-AS, **CTBP1**, CTBP1-AS2*
	chr4p12	24856	26,667	0,000	0,0055	*GABRB1*
	chr2q11.2	273834	33,333	2,778	0,0063	*TSGA10, C2orf15, LIPT1, MITD1, MRPL30, LYG2, LYG1, TXNDC9, EIF5B*
	chr3q26.33	314951	33,333	2,778	0,0063	*TTC14, CCDC39, LOC101928882*
	chr4p12	646737	33,333	2,778	0,0063	*GABRA2, COX7B2, GABRA4*
	chr4q22.1	265109	33,333	2,778	0,0063	*GRID2*
	chr11p11.2	222115	66,667	25,000	0,0098	*DGKZ, MIR4688, MDK, CHRM4, MIR3160–1, MIR3160–2, AMBRA1*

Data are number, unless otherwise indicated (%); <1= = patients who achieved CR after induction therapy; <0= = patients who achieved PR and/or SD after induction therapy; genes with * resulted differentially expressed, either down- or over-regulated, in a comparison among CR vs. PR and/or SD patients, carrying the correspondent CNA (p<0.05); in bold are indicated tumour suppressor genes.

In order to verify whether the genomic aberrations also affected gene expression, we compared the gene profiles of CR patients carrying recurrent chromosomal aberrations vs. PR/SD patients not carrying any. We showed that none of the CN gains seemed to affect the expression of the amplified genes located within the amplified region in CR patients; on the other hand, the CN losses might account for the 39% decreased expression of the genes located on the deleted regions in CR patients, half of which proved to be significantly down-expressed, with FC ranging from −0.21 to −1.29. *EVI5*, being deleted in 60% of CR patients, was also the most significant down-expressed gene (FC = −1.29, *p* = 0.00014) of those located within the 1.3Mb deletion on chr.1p22.1; the same region also included *GLMN* and *GFI1*, both significantly de-regulated in CR patients (FC = −0.46 and 0.65, respectively; *p* = 0.001 and 0.05, respectively). Of note, comparison of the above-described transcription profiles between CR and SD/PD patients showed that two out of the 52 differentially expressed genes located on chromosome 1, i.e. *BCL10* and *DPYD*, were included within the chromosome 1p22 deletion here described; their position on the chromosome is respectively telomeric and centromeric with respect to *EVI5* (Figure 1).

Of the LOH events that significantly characterized CR patients, only one, located on chr.4p16.1 and spanning 1.2Mb, included - among the other genes located within this region - a putative tumour suppressor gene, *CTBP1*, which acts as a transcriptional co-repressor in several human cancers [27]. This gene is significantly down-regulated in CR, as compared to SD/PD patients. Note that the same cytoband is known to be involved in one of the most frequent translocations described in MM, i.e. t(4;14) (p16;q32), though breakpoints on chromosome 4 have been described as being more centromeric than the above-described LOH event, falling within gene *WHSC1*, located on chr.4p16.3 [28].

## DISCUSSION

The aim of this study was to provide an expression signature to be used to predict the attainment of CR in newly diagnosed MM patients treated up-front with VTD induction therapy. In addition, we have extensively and thoroughly described the genetic and the genomic background of plasma cells obtained at baseline from patients highly sensitive to VTD front-line therapy.

### Definition of a 5-gene signature predictive of CR to VTD

We found that achievement of CR after primary induction therapy was an early predictor of prolonged PFS after ASCT and retained independent prognostic relevance in a multivariate regression analysis. The triplet VTD resulted in significant tumour shrinking in about a quarter of newly diagnosed, ASCT-eligible, MM patients. This finding suggests that, at the onset of the disease, there already exists a privileged subgroup of patients who, irrespective of the most common clinical baseline characteristics, takes the maximum benefit from this type of combination therapy and achieves CR. The possibility of predicting an optimal response to therapy is the essence of targeted-therapy and enables one to select those patients who might mostly benefit from a particular treatment. This is particularly important in MM, since it is a genetically heterogeneous disease, where peculiar genomic backgrounds characterize sub-groups of patients, or even sub-clones within the same patient's tumour.

The baseline transcriptome analysis has the advantage of capturing the overall expression profile of the neoplastic clone (or clones), thus reflecting the status of plasma cells before any interference by therapy [29]. The bio-statistical approach employed to achieve this objective needs to be rigorous and reproducible and it should aim to obtain signatures that are both highly performing and low dimensional enough to be employed in common clinical practice (i.e. in the order of 10^1^ probes). We focused on this issue by performing discriminant-like analyses on two-dimensional spaces and subsequently merging the results obtained into optimal low-dimensional signatures, with every step controlled by robust cross-validation (i.e. Leave-One-Out and k-fold cross-validation) [30, 31]. This highly robust procedure produced a 5-gene signature able both to correctly predict optimum response to VTD induction in 85% of the patients and to retain a high predictive power when tested on an independent dataset (79% and 72% in the IFMII and the APEX/SUMMIT datasets, respectively). Note that, although validation of the signature on these different datasets might have been slightly impaired by the fact that the same chip was not used to produce the transcriptome profiles (IFM II dataset), nor were patients treated with exactly the same combination (though, it was always based on the use of bortezomib), performances were anyway higher than previously reported on the same data [32]. This highlights the power of the statistical algorithm adopted in our study, arguing the idea that a gene expression profile alone might be inadequate in predicting the response to therapy [32]. Thus, the predictive power of our statistically defined signature is high enough to recommend it be employed for selection of newly diagnosed MM patients who might achieve CR with VTD induction therapy. One should note that the observed over-estimation of CR (PPV = 44%, at best) would be clinically acceptable, since it would not preclude the possibility of switching from one therapy to another, should a patient perform badly.

### GEP analysis

As well as correctly identifying, already at diagnosis, those patients who achieve CR with VTD induction therapy, it was our objective to describe the molecular characteristics of this particular subgroup of patients, in order to understand the basis of their high sensitivity to this triplet combination. No conventional baseline clinical features selectively characterize CR patients; similarly, no so far described genetic lesions, as detected by FISH analysis, have been shown to univocally correlate with response to this three-drug regimen. An imbalanced, but not significant, distribution of chromosomal aberrations (i.e. del(17p) and/or t(4;14)(p16;q32)) has been observed among patients stratified according to their response rate after VTD.

We thus employed high throughput molecular tools in order to go deeper into this issue and were able to show that patients who responded to VTD induction therapy by achieving CR actually displayed both a peculiar transcription profile and a typical genomic background. Several pathways proved significantly deregulated in CR as compared to PR/SD patients, as a consequence of activation of the different transcriptional programs in plasma cells observed at diagnosis. Broadly speaking, two apparently contrasting plasma cell phenotypes emerged: on the one hand, baseline plasma cells sensitive to VTD therapy seemed to display a highly proliferative phenotype, sustained also by the inhibition of pro-apoptotic pathways; on the other hand, an attenuated expression of pathways known to negatively impact on the cross-talk between the MM plasma cells and the microenvironment could be detected. Of the genes most significantly deregulated, *CCND1* and *CCND2* proved to be mutually exclusive in CR patients, where *CCND1* was the more down regulated, *CCND2* the more over-expressed gene. These two genes have a critical role in MM biology [33, 34]: a restricted expression of just one D cyclin in each MM case has been demonstrated and cyclin D2 has been shown to be a critical determinant for cell cycle deregulation and MM progression [34]. Hence, marked de-regulation of these genes may either be responsible for, or contribute to, the observed alteration of pathways relating to cell cycle control, apoptosis, and Wnt signalling regulation. In plasma cells obtained from CR patients, apoptosis might also be impaired because of down-regulation of the two pro-apoptotic proteins BAD and BAX, whereas *DKK1* Wnt antagonist down-regulation strongly suggests impaired Wnt signalling [35]. One notes that impaired cell cycle control and apoptosis signalling both point to an aggressive plasma cell phenotype obtained from CR patients; by contrast, the down-modulation of Wnt signalling outlines a milder scenario, where lower levels of DKK1 might lessen the possibility of osteolytic lesion formation [36]. Pathway analysis of gene profiles characterizing CR plasma cells also showed that in general the main MM plasma cell growth factor signalling cascades (i.e. NFkB, IL6R, VEGF and IGF1R signalling pathways) were differently affected in CR than in PR/SD samples, as a result of deregulation of several genes common to these strictly interconnected signalling cascades. Indeed, altered expression of key regulators of these pathways (e.g. *IGF1R*, *VEGFB*, *IL6ST*), as well as of specific factors (oncogene, tumour suppressor genes, transcription factors, e.g. *AKT2, KRAS, PTEN, FOXO3A*) might lead one to expect activation of NFkB, IL6R and IGF1R pathways, along with down-modulation of the VEGF pathway.

We thus suggest that the two observed coexisting aspects of MM plasma cell biology might account for the CR patients' tendency after VTD induction therapy to go through highly efficient clone de-bulking and a decreased interchange between plasma cells and the local *milieu*. Indeed, both these features might lead to establishment of the CR clinical phase (as assessed by conventional methods [37]), where the major clone (presumably the more proliferative ones) has been shrunk, whereas several putative minor clones (i.e. the minimal residual disease) might persist and display a milder phenotype, consistent with prolonged disease control, as commonly observed in patients who achieve CR.

### SNP analysis

A comparable dual scenario emerged from whole genome analysis of genomic CNAs by means of SNP array karyotype re-construction. The CNA most commonly shared by CR patients is CN loss on chromosome 1p22.1, which includes at least three significantly de-regulated genes. Deletions on chromosome 1p have been described as common recurrent genetic events in MM, with prognostic significance [38, 39]. In particular, the chromosomal 1p22.1–1p21–3 region represents one of the 4 minimally altered ones - on chromosome 1p - which have been described in MM [40] and are reported to be slightly associated with impaired OS or PFS in patients treated with either conventional or thalidomide-based therapy followed by ASCT. The two de-regulated genes (*MTF2* and *TMED5*) located within this region have not been reported as carrying any point mutation, which partly invalidates them as candidate genes for this chromosomal aberration [38]. Here we showed that a 606Kb sub-region, included within the 1p22.1-1p21-3 deletion – not including *MTF2* and *TMED5* - proved significantly more recurrent in CR samples: of the 20 genes covered by this CN loss, three were significantly deregulated in CR patients, which carried this deletion, i.e. *EVI5* and *GLMN*, which were down-regulated and *GFI1*, which was over-expressed. *EVI5* has been described as being involved in both cell cycle and cell migration regulation: in particular it has a role in the completion of cytokinesis and the safeguarding of genomic integrity during cell division; thus, silencing of *Evi5* resulted in cell-cycle arrest and mitotic catastrophe [41]. Both *GLMN* and *GIF1* were slightly – even if significantly – de-regulated; *GLMN* is a cullin ring ligase (CRL) inhibitor, which has recently been described as having a role in ubiquitination [42, 43]; one should note that loss of *GLMN* finally results in inefficient degradation of Cyclin E and c-Myc, which in turn leads to genomic instability and cancerogenesis [43]. *GIF1* is a DNA-binding transcriptional repressor protein with an important role in several hematopoietic lineages; it particularly affects the correct development and function of both B- and T-lymphocytes, by interaction with a number of histone-modifying enzymes [44]. None of these genes have yet been described as having a specific role in MM, even though their functions, as well as their de-regulation in CR samples carrying 1p22.1 deletion, are in line with the idea that either more aggressive or more proliferative MM phenotypes are frequently more therapy-sensitive, thus leading to the achievement of high quality clinical responses.

On the other hand, plasma cells obtained from CR patients frequently also harbour an LOH on chromosome 4p16 spanning a region carrying *CTBP1*, which proves significantly down-regulated in CR patients. This transcriptional co-repressor has been reported to be essential for cell proliferation and cell survival, as being a negative regulator of important tumour suppressor genes; moreover the down-regulation of it, mediated by some tumour suppressor, has been shown to result in p53-independent apoptosis and reduced tumour cell migration and invasion [27].

## CONCLUSION

In conclusion, we have shown that newly diagnosed MM patients may be accurately stratified according to their sensitivity to VTD induction therapy by means of a 5-gene expression signature. We also showed that patients who achieve CR after VTD carried at baseline plasma cells displaying two opposite phenotypes, both possibly contributing to the sensitivity to VTD induction therapy. We might speculate that the plasma cells we analysed at diagnosis were a heterogeneous population, i.e. a mixture of cells deriving from different clones co-existing in the same patient. This hypothesis might be consistent with recent theories on clonal evolution [45–48], and might actually suggest that these apparently contrasting behaviours reflected the presence of different sub-clones, major or minor, co-existing at diagnosis in the same patients, and fluctuating under the selective pressures exerted by the therapy. In this perspective, our results support the current multi-phase therapeutic strategies employed in MM, suggesting that front-line tumour shrinking to the point of minimal residual disease, while being an important step towards disease control, is not sufficient alone to preserve from relapses: residual disease, even if clinically undetectable, requires continuous control, aimed at delaying disease recurrence as much as possible.

## MATERIALS AND METHODS

### Patients

One hundred and eighteen patients aged 65 years or younger, with previously untreated symptomatic MM, were included in the present study based on the availability of adequate biological samples taken at diagnosis. These patients were part of the entire cohort of 236 patients who were randomly assigned to the VTD arm of the GIMEMA-MMY-3006 study (ClinicalTrials gov. number NCT01134484, EudraCT number 2005-003723-39) [8]. Induction therapy comprised three 21-day cycles of intravenous bortezomib, 1.3 mg/m^2^ on days 1, 4, 8 and 11, thalidomide, 100 mg daily for the first 14 days and 200 mg daily thereafter, plus dexamethasone, 40 mg on the day of and the day after each bortezomib infusion. Baseline clinical and prognostic variables of these 118 patients were comparable with those of the general population of patients randomly assigned to receive VTD induction therapy, with the single exception of bone marrow plasma cell infiltration, which was higher in the cohort of molecularly analysed patients (Table 5).

**Table 5:** Demographic and disease characteristics of patients at baseline

	VTD (*n* = 236)	VTD with genomic data (*n* = 118)	*p*
**Age (years)** median (IQR) mean (SD)	58.0 (52.0–62.0) 56.3 (6.9)	58.1 (53.7–62.3) 57.0 (6.8)	ns ns
**Sex** Male Female	137 (58%) 99 (42%)	75 (64%) 43 (36%)	ns
**Myeloma subtype** IgG IgA Light chain other	154 (65%) 41 (17%) 40 (17%) 1 (<1%)	81 (69%) 19 (16%) 18 (15%) 0 (<1%)	ns
**ISS disease stage** I II III	107 (45%) 91 (39%) 38 (16%)	55 (47%) 43 (36%) 20 (17%)	ns
**b2-microglobulin (mg(L)** median (IQR) mean (SD)	3.0 (2.3–4.4) 3.8 (2.5)	3.0 (2.3–4.4) 3.8 (2.5)	ns ns
**Albumin (g/L)** median (IQR) mean (SD)	38.3 (34.0–43.2) 38.3 (6.4)	38.2 (33.0–43.0) 38.0 (6.5)	ns ns
**Creatinine (mmol/L)** median (IQR) mean (SD)	84.5 (70.4–96.8) 88.5 (26.7)	88.4 (70.7–98.1) 92.2 (28.1)	ns ns
**Haemoglobin (g/L)** median (IQR) mean (SD)	111.5 (96.0–125.0) 111.0 (19.2)	108.5 (95.0–121.0) 109.2 (18.9)	ns ns
**Platelets (×10^9^ per L)** median (IQR) mean (SD)	231.5 (187.2–286.8) 243.7 (89.3)	230.0 (186.0–294.0) 241.3 (86.8)	ns ns
**Bone Marrow Plasma Cells** median (IQR) mean (SD)	50 (35–70) 52.4 (23.2)	60 (45–75) 59.4 (19.9)	<0.0001** <0.0001**
Cytogenetic abnormalities* presence of del (13q) presence of t(4;14)(p16;q32) presence of del(17p)	103 (47%) 41 (19%) 15 (7%)	59 (51%) 21 (18%) 9 (8%)	ns ns ns

Data are number (%), unless otherwise indicated. VTD = bortezomib with thalidomide and dexamethasone. ISS = International staging system

* 218 patients were available for assessment.

** highly significant

### Response definition

Criteria for evaluating response to therapy were as reported by the European Group for Blood and Marrow Transplantation [49], with the addition of nCR (100% reduction in M protein according to electrophoresis, but immunofixation positive) [50] and VGPR (≥90% reduction in serum M protein, and less than 100 mg urine M protein per day) categories [37].

### Sample collection, CD138+ cell fraction enrichment, data generation and quality control

Bone marrow (BM) samples for molecular studies were obtained during standard diagnostic procedures, after a written informed consent was provided by each patient. Plasma cells were purified from mononuclear BM cells obtained by Ficoll-Hypaque density gradient centrifugation using anti-CD138 micro beads on an AutoMacs Magnetic Cell Separator (MACS system, Miltenyi Biotec, Auburn, CA). The purity of positively selected plasma cells was ≥90% in all cases. Total genomic DNA was extracted using a Maxwell^®^ 16 LEV Blood DNA kit (Promega, Madison, WI). To measure the concentration and purity of both nucleic acids, a NanoDrop ND-1000 spectrophotometer was used (NanoDrop Technologies, Wilmington, DE). Total RNA was obtained from each sample by the RNeasy^®^ kit (Qiagen, Valencia, CA) extraction procedure: the RNeasy^®^ Mini kit was used for more than 5 × 10^5^ cells, the RNeasy^®^ Micro kit for less than 5 × 10^5^ cells.

Total RNA from purified samples was labelled for gene expression profiling starting from 100 ng, by means of the Affymetrix Two-cycle Gene Chip microarray system. Preparation of DNA single-stranded sense target, hybridization to HG U133 Plus 2.0 GeneChip arrays, and scanning of the arrays were performed according to the manufacturer's protocols (Affymetrix, Santa Clara, CA). Gene expression CEL files are available for free download at http://www.ncbi.nlm.nih.gov (GEO, Gene Expression Omnibus), accession number GSE69029, subseries GSE68871.

In 89 patients out of 118 for whom at least 500 ng DNA was available, SNP array experiments were performed, using 6.0 SNP-based mapping Genome-wide Human GeneChip (Affymetrix, Santa Clara, CA); fluorescence data were acquired by a scanning procedure and translated into a CEL file. The Toronto Database of Genomic Variants was used to compute accurate copy number alteration analysis of primary tumour cells. SNPs CEL files are available for free download at http://www.ncbi.nlm.nih.gov (GEO), accession number GSE69029, subseries GSE69028.

### Statistical analysis

To determine the differentially expressed genes between patients who either achieved or failed CR after VTD induction therapy, the Affymetrix output (CEL files) were processed by Affymetrix Power Tools with Robust Multi-Array (RMA) normalization. Quality controls revealed that no sample needed to be discarded for poor quality. Samples were further normalized at a global level by quantile normalization (Mathworks Matlab software) for subsequent analysis. Differentially expressed genes between groups with different responses to VTD induction therapy were then analysed by means of GeneGo^®^ software.

To obtain a low-dimensional gene-based classifier able to discriminate between CR and non-CR patients on the basis of their transcription profile, an original method - based on Fisher Quadratic Discriminant Analysis - was employed. This method proved able to obtain optimal prediction performances in terms of accuracy. The designed algorithm first compared several small subsets of probe sets (i.e. all the probe couples trained with Leave-One-Out cross-validation). From the top-scoring couples (in terms of classification performance of the dataset) various different signatures were obtained by means of a network approach (combining best couples into networks): the best performing signature was chosen. The optimal signature was tested on the available dataset by 3-fold cross-validation repeated 1000 times (thus randomly dividing the full dataset into 3 parts, two for training and one for testing): this generated an exhaustive picture of the overall signature performance, with a maximum, minimum and median performance over 1000 realizations.

Affymetrix Genotyping Console (GC) was used to extract the raw DNA copy number (CN) and loss-of-heterozygosity (LOH) probability and to evaluate quality parameters of CEL files according to Affymetrix guidelines. CEL files were then loaded into Nexus Copy Number software (professional version 7.5, Biodiscovery, Hawthorne, CA), which allowed a number of samples to be analyzed simultaneously, in order to identify common areas of chromosome aberrations. Data where finally processed with the BioDiscovery's Rank Segmentation algorithm, thus generating a frequency plot. In order to identify statistically significant differences between sample subgroups, we used the “comparisons” feature of Nexus, which identified the CNAs whose frequencies proved significantly different when comparing various subgroups of patients. Fisher's exact test was used to calculate the *p*-values and the False Discovery Rate (FDR) adjustment was used to correct for multiple testing. The most significant regions of copy number changes were annotated.

## SUPPLEMENTARY TABLE




