# Supplementary material for: The genetic and genomic background of multiple myeloma patients achieving complete response after induction therapy with bortezomib, thalidomide and dexamethasone (VTD)
Source: Oncotarget. 2015 Nov 9;7(9):9666–79. doi: 10.18632/oncotarget.5718 (PMC4891075; doi:10.18632/oncotarget.5718)
Supplement: Supplementary file 1 [file oncotarget-07-09666-s001.pdf]

SUPPLEMENTARY TABLE

Supplementary Table S1: list of probe sets significantly differentially expressed in CR vs NR patients, filtered according to the GeneGo® "haematological disease" category
