# Supplementary material for: The genetic and genomic background of multiple myeloma patients achieving complete response after induction therapy with bortezomib, thalidomide and dexamethasone (VTD)
Source: Oncotarget. 2015 Nov 9;7(9):9666–79. doi: 10.18632/oncotarget.5718 (PMC4891075; doi:10.18632/oncotarget.5718)
Supplement: Supplementary file 2 [file oncotarget-07-09666-s002.doc]

| **ID** | **Symbol** | **p-value** | **Log Ratio** |
| --- | --- | --- | --- |
| 1565082_x_at | SRC | 2,46E-05 | -0,283 |
| 214108_at | MAX | 1,04E-04 | -0,692 |
| 239853_at | KLC3 | 1,26E-04 | -0,311 |
| 200026_at | RPL34 | 1,87E-04 | -0,383 |
| 209951_s_at | MAP2K7 | 2,63E-04 | -0,355 |
| 230690_at | TUBB1 | 2,70E-04 | -1,14 |
| 208639_x_at | PDIA6 | 3,09E-04 | 0,547 |
| 1564130_x_at | QPRT | 3,55E-04 | -0,196 |
| 206390_x_at | PF4 | 3,81E-04 | -1,377 |
| 203333_at | KIFAP3 | 4,36E-04 | 0,771 |
| 201042_at | TGM2 | 4,47E-04 | -0,377 |
| 203085_s_at | TGFB1 | 4,95E-04 | -0,903 |
| 200953_s_at | CCND2 | 6,12E-04 | 3,303 |
| 1569410_at | FLG2 | 6,61E-04 | -0,172 |
| 201724_s_at | GALNT1 | 6,86E-04 | 0,945 |
| 235495_at | CCDC97 | 6,86E-04 | -0,254 |
| 210049_at | SERPINC1 | 6,88E-04 | -0,16 |
| 1554508_at | PIK3AP1 | 7,01E-04 | -0,305 |
| 1556017_at | NBEAL2 | 8,15E-04 | -0,304 |
| 218259_at | MKL2 | 8,68E-04 | 0,688 |
| 213987_s_at | CDK13 | 9,38E-04 | -0,481 |
| 209808_x_at | ING1 | 9,64E-04 | -0,393 |
| 215955_x_at | ARHGAP26 | 1,02E-03 | -0,271 |
| 242282_at | ZFPM1 | 1,23E-03 | -0,601 |
| 226195_at | IFT43 | 1,30E-03 | -0,577 |
| 1553740_a_at | IRAK2 | 1,31E-03 | -0,199 |
| 204450_x_at | APOA1 | 1,40E-03 | -0,276 |
| 201389_at | ITGA5 | 1,42E-03 | -0,258 |
| 207849_at | IL2 | 1,51E-03 | -0,135 |
| 219942_at | MYL7 | 1,66E-03 | -0,211 |
| 211074_at | FOLR1 | 1,85E-03 | -0,945 |
| 1553042_a_at | NFKBID | 1,99E-03 | -0,3 |
| 212191_x_at | RPL13 | 2,16E-03 | -0,817 |
| 203775_at | SLC25A13 | 2,20E-03 | 0,591 |
| 208979_at | NCOA6 | 2,20E-03 | 0,391 |
| 213040_s_at | NPTXR | 2,22E-03 | -0,213 |
| 232881_at | GNAS-AS1 | 2,30E-03 | 0,162 |
| 228830_s_at | ATF7 | 2,32E-03 | -0,737 |
| 232727_at | UCKL1 | 2,32E-03 | -0,225 |
| 213614_x_at | EEF1A1 | 2,33E-03 | 0,181 |
| 206655_s_at | GP1BB | 2,35E-03 | -0,526 |
| 228518_at | IGHM | 2,40E-03 | -2,656 |
| 204463_s_at | EDNRA | 2,58E-03 | -0,121 |
| 201753_s_at | ADD3 | 2,59E-03 | 1,058 |
| 206209_s_at | CA4 | 2,65E-03 | -0,385 |
| 206267_s_at | MATK | 2,78E-03 | -0,336 |
| 203783_x_at | POLRMT | 2,79E-03 | -0,297 |
| 206669_at | GAD1 | 2,88E-03 | -0,238 |
| 1567906_at | SOX4 | 2,90E-03 | -0,236 |
| 236686_at | MVK | 2,93E-03 | -0,207 |
| 223046_at | EGLN1 | 3,01E-03 | 0,697 |
| 210173_at | PTPRJ | 3,04E-03 | -0,156 |
| 200091_s_at | RPS25 | 3,12E-03 | -0,31 |
| 213774_s_at | PPP1R2 | 3,14E-03 | -0,169 |
| 201616_s_at | CALD1 | 3,17E-03 | -0,304 |
| 207550_at | MPL | 3,29E-03 | -0,306 |
| 211835_at | IGKC | 3,34E-03 | -0,462 |
| 214673_s_at | HUWE1 | 3,35E-03 | -0,236 |
| 208461_at | HIC1 | 3,38E-03 | -0,34 |
| 226103_at | NEXN | 3,49E-03 | -0,217 |
| 227471_at | HACE1 | 3,51E-03 | 0,695 |
| 236926_at | TBX1 | 3,54E-03 | -0,351 |
| 1558631_at | PPARA | 3,59E-03 | -0,213 |
| 216836_s_at | ERBB2 | 3,73E-03 | -0,403 |
| 216632_at | NAV3 | 3,92E-03 | -0,149 |
| 209666_s_at | CHUK | 3,93E-03 | 0,633 |
| 213644_at | CEP112 | 3,97E-03 | 0,564 |
| 1553545_at | ILDR1 | 4,03E-03 | -0,194 |
| 203643_at | ERF | 4,07E-03 | -0,69 |
| 215461_at | ZNRF4 | 4,14E-03 | -0,301 |
| 208501_at | GFI1B | 4,40E-03 | -0,296 |
| 211359_s_at | OPRM1 | 4,40E-03 | 0,282 |
| 1562330_s_at | CSMD1 | 4,42E-03 | -0,136 |
| 218002_s_at | CXCL14 | 4,44E-03 | -0,312 |
| 201065_s_at | GTF2I | 4,45E-03 | 0,542 |
| 204678_s_at | KCNK1 | 4,53E-03 | -0,305 |
| 1566722_a_at | SVEP1 | 4,62E-03 | -0,117 |
| 1552803_a_at | STMN1 | 4,64E-03 | -0,214 |
| 215122_at | TBX6 | 4,77E-03 | -0,165 |
| 230628_at | EP400 | 4,83E-03 | -0,288 |
| 226191_at | GSK3B | 5,19E-03 | 0,494 |
| 221672_s_at | TRAPPC9 | 5,40E-03 | -0,311 |
| 209093_s_at | GBA | 5,87E-03 | 0,455 |
| 217266_at | RPL15 | 5,90E-03 | -0,413 |
| 201665_x_at | RPS17 | 5,94E-03 | -0,215 |
| 205418_at | FES | 6,01E-03 | -0,415 |
| 234166_at | UBE3A | 6,01E-03 | -0,137 |
| 203508_at | TNFRSF1B | 6,08E-03 | -0,557 |
| 200010_at | RPL11 | 6,15E-03 | -0,787 |
| 211430_s_at | IGHG1 | 6,18E-03 | -2,018 |
| 210158_at | ERCC4 | 6,36E-03 | 0,256 |
| 203683_s_at | VEGFB | 6,38E-03 | -0,407 |
| 200651_at | GNB2L1 | 6,43E-03 | -0,483 |
| 201479_at | DKC1 | 6,50E-03 | 0,847 |
| 1557994_at | TTN | 6,52E-03 | -0,137 |
| 211087_x_at | MAPK14 | 6,67E-03 | -0,228 |
| 229935_s_at | KMT2A | 6,89E-03 | -0,168 |
| 227681_at | ZFP36L2 | 6,90E-03 | -0,218 |
| 201598_s_at | INPPL1 | 6,96E-03 | -0,275 |
| 41657_at | STK11 | 7,28E-03 | -0,28 |
| 207389_at | GP1BA | 7,39E-03 | -0,269 |
| 220662_s_at | HEYL | 7,39E-03 | -0,228 |
| 242451_x_at | RPS19 | 7,39E-03 | -0,306 |
| 1552507_at | KCNE4 | 7,41E-03 | -0,154 |
| 223303_at | FERMT3 | 7,60E-03 | -0,376 |
| 212237_at | ASXL1 | 7,72E-03 | 0,401 |
| 203633_at | CPT1A | 7,76E-03 | -0,825 |
| 200736_s_at | GPX1 | 7,78E-03 | -0,754 |
| 208377_s_at | CACNA1F | 7,88E-03 | -0,258 |
| 206689_x_at | KAT5 | 8,00E-03 | -0,335 |
| 213337_s_at | SOCS1 | 8,07E-03 | -0,263 |
| 219186_at | ZBTB7A | 8,10E-03 | -0,537 |
| 1558546_at | DNASE1 | 8,33E-03 | -0,28 |
| 1567013_at | NFE2L2 | 8,46E-03 | -0,359 |
| 214139_at | ARID4B | 8,46E-03 | -0,355 |
| 206283_s_at | TAL1 | 8,53E-03 | -0,58 |
| 214378_at | TFPI | 8,53E-03 | -0,189 |
| 212066_s_at | USP34 | 8,57E-03 | 0,467 |
| 210423_s_at | SLC11A1 | 8,61E-03 | -0,458 |
| 229438_at | FAM20C | 8,69E-03 | -0,203 |
| 216845_x_at | KMT2D | 8,71E-03 | -0,216 |
| 203238_s_at | NOTCH3 | 8,74E-03 | -0,294 |
| 208074_s_at | AP2S1 | 8,81E-03 | -0,588 |
| 229450_at | IFIT3 | 8,84E-03 | -1,617 |
| 219510_at | POLQ | 8,87E-03 | 0,497 |
| 208567_s_at | KCNJ12 | 8,92E-03 | -0,144 |
| 212610_at | PTPN11 | 9,05E-03 | 0,374 |
| 211904_x_at | RAD52 | 9,06E-03 | -0,225 |
| 220549_at | RAD54B | 9,18E-03 | 0,506 |
| 228133_s_at | MYH11 | 9,23E-03 | -0,434 |
| 244782_at | PPP3R2 | 9,27E-03 | -0,107 |
| 208860_s_at | ATRX | 9,48E-03 | 0,463 |
| 218926_at | MYNN | 9,62E-03 | 0,748 |
| 206494_s_at | ITGA2B | 9,80E-03 | -0,427 |
| 210229_s_at | CSF2 | 9,87E-03 | -0,243 |
| 227072_at | RTTN | 9,90E-03 | 0,607 |
| 1560396_at | KLHL6 | 9,91E-03 | -0,659 |
| 201058_s_at | MYL9 | 1,01E-02 | -0,25 |
| 207206_s_at | ALOX12 | 1,01E-02 | -0,274 |
| 208629_s_at | HADHA | 1,02E-02 | -0,746 |
| 202854_at | HPRT1 | 1,03E-02 | 0,718 |
| 201198_s_at | PSMD1 | 1,04E-02 | 0,388 |
| 202921_s_at | ANK2 | 1,04E-02 | 0,18 |
| 205039_s_at | IKZF1 | 1,05E-02 | -0,802 |
| 1566208_at | TCEA1 | 1,06E-02 | -0,141 |
| 1554069_at | EPHA8 | 1,07E-02 | -0,159 |
| 1861_at | BAD | 1,08E-02 | -0,692 |
| 220937_s_at | ST6GALNAC4 | 1,08E-02 | -0,522 |
| 226281_at | DNER | 1,09E-02 | -0,135 |
| 217005_at | LDLR | 1,10E-02 | -0,172 |
| 221271_at | IL21 | 1,10E-02 | 0,163 |
| 211654_x_at | HLA-DQB1 | 1,11E-02 | -0,529 |
| 234253_at | MYCN | 1,11E-02 | 0,219 |
| 213606_s_at | ARHGDIA | 1,12E-02 | -0,705 |
| 217070_at | MTHFR | 1,12E-02 | -0,213 |
| 207670_at | KRT85 | 1,13E-02 | -0,218 |
| 227422_at | STRN | 1,15E-02 | 0,594 |
| 230237_at | ADCYAP1 | 1,15E-02 | -0,103 |
| 210045_at | IDH2 | 1,16E-02 | -0,648 |
| 228904_at | HOXB3 | 1,16E-02 | 0,927 |
| 208441_at | IGF1R | 1,17E-02 | 0,161 |
| 203990_s_at | KDM6A | 1,18E-02 | -0,25 |
| 204880_at | MGMT | 1,18E-02 | -0,675 |
| 236263_at | SHH | 1,18E-02 | -0,143 |
| 49049_at | DTX3 | 1,18E-02 | -0,218 |
| 210416_s_at | CHEK2 | 1,19E-02 | 0,651 |
| 200640_at | YWHAZ | 1,20E-02 | 0,526 |
| 229482_at | DDX51 | 1,20E-02 | -0,285 |
| 207847_s_at | MUC1 | 1,22E-02 | 0,543 |
| 217466_x_at | RPS2 | 1,22E-02 | -0,588 |
| 206126_at | CXCR5 | 1,23E-02 | -0,364 |
| 201084_s_at | BCLAF1 | 1,24E-02 | 0,503 |
| 206883_x_at | GP9 | 1,25E-02 | -0,197 |
| 217298_at | RPS14 | 1,25E-02 | -0,182 |
| 226908_at | LRIG3 | 1,25E-02 | 0,812 |
| 231662_at | ARG1 | 1,25E-02 | -0,109 |
| 204428_s_at | LCAT | 1,28E-02 | -0,391 |
| 206219_s_at | VAV1 | 1,29E-02 | 0,618 |
| 202911_at | MSH6 | 1,30E-02 | 0,473 |
| 210031_at | CD247 | 1,30E-02 | -0,327 |
| 229600_s_at | CPD | 1,31E-02 | -0,225 |
| 209468_at | LRP5 | 1,34E-02 | -0,292 |
| 231359_at | APOH | 1,35E-02 | -0,142 |
| 208023_at | TNFRSF4 | 1,36E-02 | -0,169 |
| 202205_at | VASP | 1,37E-02 | -0,569 |
| 209134_s_at | RPS6 | 1,37E-02 | -0,129 |
| 223556_at | HELLS | 1,38E-02 | 0,845 |
| 209136_s_at | USP10 | 1,39E-02 | -0,506 |
| 37831_at | SIPA1L3 | 1,40E-02 | -0,334 |
| 204639_at | ADA | 1,41E-02 | 0,55 |
| 211492_s_at | ADRA1A | 1,41E-02 | -0,14 |
| 1555006_at | WDR66 | 1,42E-02 | 0,26 |
| 1553534_at | NLRP10 | 1,44E-02 | -0,163 |
| 207400_at | NPY5R | 1,44E-02 | -0,151 |
| 230437_s_at | PRKCB | 1,44E-02 | 0,191 |
| 208196_x_at | NFATC1 | 1,45E-02 | -0,225 |
| 204694_at | AFP | 1,46E-02 | -0,189 |
| 206758_at | EDN2 | 1,46E-02 | -0,151 |
| 211568_at | BAI3 | 1,46E-02 | -0,215 |
| 222735_at | TMEM38B | 1,46E-02 | 0,479 |
| 212934_at | UBXN2B | 1,47E-02 | 0,566 |
| 213971_s_at | SUZ12 | 1,47E-02 | -0,463 |
| 213980_s_at | CTBP1 | 1,47E-02 | -0,622 |
| 221722_x_at | LZTS1 | 1,47E-02 | -0,288 |
| 1553174_at | JPH2 | 1,48E-02 | -0,227 |
| 1567628_at | CD74 | 1,49E-02 | -0,249 |
| 209804_at | DCLRE1A | 1,50E-02 | 0,572 |
| 204406_at | FLT1 | 1,52E-02 | -0,111 |
| 234408_at | IL17F | 1,52E-02 | -0,176 |
| 200606_at | DSP | 1,53E-02 | -0,239 |
| 211616_s_at | HTR2A | 1,54E-02 | -0,201 |
| 213330_s_at | STIP1 | 1,56E-02 | -0,355 |
| 223025_s_at | AP1M1 | 1,56E-02 | -0,238 |
| 235578_at | ABCC9 | 1,56E-02 | -0,126 |
| 207278_s_at | CD209 | 1,57E-02 | 0,237 |
| 1552892_at | TNFRSF13C | 1,59E-02 | -0,399 |
| 200002_at | RPL35 | 1,59E-02 | -0,327 |
| 212884_x_at | APOE | 1,59E-02 | -0,409 |
| 203305_at | F13A1 | 1,60E-02 | -0,606 |
| 225406_at | TWSG1 | 1,60E-02 | 0,567 |
| 205875_s_at | TREX1 | 1,63E-02 | -0,449 |
| 222164_at | FGFR1 | 1,64E-02 | -0,295 |
| 201302_at | ANXA4 | 1,66E-02 | 0,764 |
| 241499_at | ZNF621 | 1,66E-02 | -0,116 |
| 213578_at | BMPR1A | 1,67E-02 | 0,924 |
| 1554519_at | CD80 | 1,68E-02 | 0,191 |
| 204534_at | VTN | 1,68E-02 | -0,183 |
| 225447_at | GPD2 | 1,68E-02 | 0,585 |
| 240278_at | RASSF1 | 1,68E-02 | -0,468 |
| 204018_x_at | HBA1/HBA2 | 1,73E-02 | -1,559 |
| 206069_s_at | ACADL | 1,73E-02 | -0,115 |
| 218451_at | CDCP1 | 1,75E-02 | -0,185 |
| 240528_s_at | EXOC4 | 1,75E-02 | 0,401 |
| 56748_at | TRIM10 | 1,75E-02 | -0,243 |
| 235885_at | P2RY12 | 1,76E-02 | -0,228 |
| 244790_at | MTCP1 | 1,76E-02 | 0,491 |
| 205558_at | TRAF6 | 1,77E-02 | 0,506 |
| 208033_s_at | ZFHX3 | 1,78E-02 | -0,286 |
| 214433_s_at | SELENBP1 | 1,78E-02 | -0,627 |
| 244538_at | KCTD19 | 1,78E-02 | 0,132 |
| 203719_at | ERCC1 | 1,79E-02 | -0,515 |
| 205063_at | GEMIN2 | 1,79E-02 | -0,7 |
| 208699_x_at | TKT | 1,79E-02 | -0,559 |
| 1555003_at | RBL1 | 1,82E-02 | 0,206 |
| 1565772_at | PAK1 | 1,83E-02 | -0,163 |
| 200933_x_at | RPS4X | 1,83E-02 | 0,125 |
| 206703_at | CHRNB1 | 1,83E-02 | -0,433 |
| 227988_s_at | VPS13A | 1,83E-02 | 0,809 |
| 203058_s_at | PAPSS2 | 1,84E-02 | 1,077 |
| 207341_at | PRTN3 | 1,84E-02 | -0,47 |
| 217856_at | RBM8A | 1,85E-02 | -0,569 |
| 220496_at | CLEC1B | 1,86E-02 | -0,357 |
| 235816_s_at | RGL4 | 1,87E-02 | -0,473 |
| 211942_x_at | RPL13A | 1,88E-02 | -0,594 |
| 208712_at | CCND1 | 1,89E-02 | -2,217 |
| 225204_at | PPTC7 | 1,89E-02 | 0,568 |
| 1552497_a_at | SLAMF6 | 1,91E-02 | 0,851 |
| 205119_s_at | FPR1 | 1,91E-02 | -0,812 |
| 200061_s_at | RPS24 | 1,92E-02 | -0,283 |
| 202426_s_at | RXRA | 1,92E-02 | -0,289 |
| 223322_at | RASSF5 | 1,92E-02 | 0,735 |
| 217430_x_at | COL1A1 | 1,96E-02 | -0,152 |
| 40149_at | SH2B1 | 1,97E-02 | -0,399 |
| 211834_s_at | TP63 | 2,00E-02 | -0,147 |
| 209081_s_at | COL18A1 | 2,01E-02 | -0,322 |
| 209116_x_at | HBB | 2,01E-02 | -1,498 |
| 200022_at | RPL18 | 2,04E-02 | -0,316 |
| 211372_s_at | IL1R2 | 2,04E-02 | -0,445 |
| 202397_at | NUTF2 | 2,05E-02 | -0,753 |
| 213635_s_at | SAFB | 2,07E-02 | -0,319 |
| 219283_at | C1GALT1C1 | 2,07E-02 | 0,499 |
| 231861_at | LRP10 | 2,07E-02 | -0,523 |
| 211762_s_at | KPNA2 | 2,08E-02 | 0,46 |
| 202241_at | TRIB1 | 2,09E-02 | 0,318 |
| 205263_at | BCL10 | 2,12E-02 | 0,468 |
| 218358_at | CRELD2 | 2,12E-02 | 0,474 |
| 204895_x_at | MUC4 | 2,13E-02 | -0,158 |
| 1559921_at | PECAM1 | 2,15E-02 | 0,537 |
| 224629_at | LMAN1 | 2,15E-02 | 0,423 |
| 205436_s_at | H2AFX | 2,16E-02 | -0,656 |
| 208169_s_at | PTGER3 | 2,16E-02 | 0,167 |
| 208413_at | RARB | 2,18E-02 | 0,107 |
| 212097_at | CAV1 | 2,19E-02 | 0,867 |
| 215726_s_at | CYB5A | 2,20E-02 | 0,816 |
| 1555763_x_at | MKL1 | 2,23E-02 | 0,408 |
| 201182_s_at | CHD4 | 2,24E-02 | -0,645 |
| 214575_s_at | AZU1 | 2,24E-02 | -0,617 |
| 204713_s_at | F5 | 2,25E-02 | -0,257 |
| 242711_x_at | FANCM | 2,27E-02 | 0,302 |
| 204835_at | POLA1 | 2,28E-02 | 0,601 |
| 220208_at | ADAMTS13 | 2,28E-02 | -0,198 |
| 240586_at | ENAM | 2,28E-02 | -0,343 |
| 1559036_at | C4A/C4B | 2,29E-02 | -0,12 |
| 221355_at | CHRNG | 2,29E-02 | -0,225 |
| 203887_s_at | THBD | 2,32E-02 | -0,354 |
| 211373_s_at | PSEN2 | 2,32E-02 | 0,697 |
| 213536_s_at | UBE2I | 2,33E-02 | 0,17 |
| 217684_at | TYMS | 2,34E-02 | -0,157 |
| 209115_at | UBA3 | 2,35E-02 | 0,404 |
| 226794_at | STXBP5 | 2,35E-02 | 0,489 |
| 207793_s_at | EPB41 | 2,36E-02 | -0,106 |
| 202639_s_at | RANBP3 | 2,37E-02 | -0,218 |
| 228006_at | PTEN | 2,37E-02 | 0,474 |
| 224899_s_at | MAGT1 | 2,38E-02 | 0,541 |
| 211524_at | NFKB2 | 2,39E-02 | 0,109 |
| 200003_s_at | RPL28 | 2,41E-02 | -0,596 |
| 203957_at | E2F6 | 2,42E-02 | 0,544 |
| 202265_at | BMI1 | 2,45E-02 | 0,409 |
| 213116_at | NEK3 | 2,47E-02 | 0,48 |
| 232811_x_at | PRICKLE1 | 2,47E-02 | -0,255 |
| 203808_at | AKT2 | 2,49E-02 | -0,138 |
| 226342_at | SPTBN1 | 2,49E-02 | 0,659 |
| 227426_at | SOS1 | 2,49E-02 | 0,532 |
| 211330_s_at | HFE | 2,52E-02 | 0,405 |
| 201469_s_at | SHC1 | 2,53E-02 | 0,325 |
| 204691_x_at | PLA2G6 | 2,53E-02 | -0,198 |
| 207577_at | HTR4 | 2,53E-02 | 0,18 |
| 214203_s_at | LOC102724788/PRODH | 2,53E-02 | -0,301 |
| 231968_at | UGGT1 | 2,55E-02 | 0,385 |
| 206211_at | SELE | 2,56E-02 | 0,112 |
| 213204_at | CUL9 | 2,56E-02 | -0,329 |
| 1569850_at | LPHN2 | 2,57E-02 | -0,336 |
| 225465_at | MAGI1 | 2,57E-02 | 0,304 |
| 206153_at | CYP4F11 | 2,59E-02 | -0,163 |
| 242352_at | NIPBL | 2,61E-02 | -1,235 |
| 243495_s_at | ZNF652 | 2,61E-02 | 1,2 |
| 226734_at | EIF4E2 | 2,62E-02 | 0,401 |
| 1568924_a_at | IQUB | 2,64E-02 | 0,12 |
| 218173_s_at | WHSC1L1 | 2,65E-02 | 0,514 |
| 232421_at | SCARB1 | 2,66E-02 | -0,155 |
| 206660_at | IGLL1/IGLL5 | 2,68E-02 | -0,205 |
| 209603_at | GATA3 | 2,68E-02 | -0,221 |
| 226619_at | SENP1 | 2,69E-02 | 0,471 |
| 208018_s_at | HCK | 2,71E-02 | -0,554 |
| 211027_s_at | IKBKB | 2,72E-02 | -0,139 |
| 230031_at | HSPA5 | 2,72E-02 | -0,538 |
| 200920_s_at | BTG1 | 2,73E-02 | -0,73 |
| 208641_s_at | RAC1 | 2,76E-02 | -0,276 |
| 219669_at | CD177 | 2,78E-02 | -0,382 |
| 204411_at | KIF21B | 2,79E-02 | 0,96 |
| 235661_at | POU2F2 | 2,79E-02 | -0,623 |
| 223481_s_at | MRPL47 | 2,80E-02 | 0,199 |
| 225289_at | STAT3 | 2,80E-02 | -0,492 |
| 216867_s_at | PDGFA | 2,82E-02 | -0,218 |
| 1554306_at | ITPKB | 2,83E-02 | -0,605 |
| 1554534_at | DPYD | 2,85E-02 | -0,155 |
| 210324_at | C8G | 2,85E-02 | -0,196 |
| 1554899_s_at | FCER1G | 2,86E-02 | -1,07 |
| 205667_at | WRN | 2,87E-02 | 0,586 |
| 203749_s_at | RARA | 2,90E-02 | -0,827 |
| 206923_at | PRKCA | 2,90E-02 | 0,141 |
| 1552611_a_at | JAK1 | 2,91E-02 | -1,245 |
| 202804_at | ABCC1 | 2,91E-02 | 0,316 |
| 204259_at | MMP7 | 2,92E-02 | -0,122 |
| 209434_s_at | PPAT | 2,96E-02 | 0,749 |
| 211013_x_at | PML | 2,96E-02 | -0,323 |
| 217026_at | CFTR | 2,96E-02 | 0,154 |
| 228813_at | HDAC4 | 2,96E-02 | 0,441 |
| 1560265_at | GRIK2 | 2,98E-02 | 0,13 |
| 215054_at | EPOR | 3,00E-02 | -0,276 |
| 228531_at | SAMD9 | 3,00E-02 | 0,743 |
| 1553678_a_at | ITGB1 | 3,01E-02 | 1,117 |
| 203052_at | C2 | 3,03E-02 | 0,931 |
| 210233_at | IL1RAP | 3,04E-02 | -0,31 |
| 213950_s_at | PPP3CC | 3,05E-02 | -0,166 |
| 221282_x_at | RUNX2 | 3,05E-02 | 0,12 |
| 203295_s_at | ATP1A2 | 3,08E-02 | 0,144 |
| 201340_s_at | ENC1 | 3,13E-02 | -0,374 |
| 205950_s_at | CA1 | 3,14E-02 | -1,025 |
| 210828_s_at | ARNT | 3,14E-02 | 0,191 |
| 223664_x_at | BCL2L13 | 3,14E-02 | -0,36 |
| 238043_at | ARID1B | 3,14E-02 | 0,519 |
| 1555713_at | PNKD | 3,15E-02 | -0,159 |
| 243475_at | CBL | 3,15E-02 | 0,181 |
| 209317_at | POLR1C | 3,17E-02 | 0,404 |
| 217091_at | FXYD2 | 3,18E-02 | -0,088 |
| 231854_at | PIK3CA | 3,18E-02 | 0,238 |
| 217262_s_at | CELSR1 | 3,20E-02 | -0,37 |
| 220498_at | ACTL7B | 3,22E-02 | -0,144 |
| 223321_s_at | FGFRL1 | 3,22E-02 | -0,241 |
| 239050_s_at | CRIPT | 3,22E-02 | 0,568 |
| 204513_s_at | ELMO1 | 3,24E-02 | -0,602 |
| 204786_s_at | IFNAR2 | 3,25E-02 | -0,346 |
| 200737_at | PGK1 | 3,26E-02 | 0,511 |
| 1558000_at | ARID5B | 3,27E-02 | -0,222 |
| 225968_at | PRICKLE2 | 3,29E-02 | 0,517 |
| 1555590_a_at | GATA1 | 3,35E-02 | -0,202 |
| 209324_s_at | RGS16 | 3,35E-02 | -0,846 |
| 214322_at | CAMK2G | 3,36E-02 | -0,205 |
| 228644_s_at | SLC12A4 | 3,36E-02 | -0,152 |
| 200082_s_at | RPS7 | 3,38E-02 | -0,711 |
| 203397_s_at | GALNT3 | 3,39E-02 | 0,632 |
| 226503_at | RIF1 | 3,39E-02 | 0,709 |
| 242061_at | RSPO2 | 3,41E-02 | -0,097 |
| 201468_s_at | NQO1 | 3,43E-02 | 0,231 |
| 204602_at | DKK1 | 3,45E-02 | -1,868 |
| 206499_s_at | RCC1 | 3,46E-02 | -0,257 |
| 210990_s_at | LAMA4 | 3,47E-02 | -0,215 |
| 1555612_s_at | G6PC | 3,48E-02 | 0,217 |
| 236749_at | MNT | 3,50E-02 | -0,149 |
| 204297_at | PIK3C3 | 3,51E-02 | 0,415 |
| 203847_s_at | AKAP8 | 3,52E-02 | 0,345 |
| 217468_at | CYP2D6 | 3,52E-02 | -0,2 |
| 201180_s_at | GNAI3 | 3,53E-02 | 0,445 |
| 202932_at | YES1 | 3,54E-02 | 0,805 |
| 204007_at | FCGR3A/FCGR3B | 3,54E-02 | -0,77 |
| 210746_s_at | EPB42 | 3,54E-02 | -0,593 |
| 49306_at | RASSF4 | 3,54E-02 | -0,192 |
| 1553544_at | GPR101 | 3,57E-02 | -0,163 |
| 204476_s_at | PC | 3,57E-02 | -0,329 |
| 211234_x_at | ESR1 | 3,57E-02 | -0,187 |
| 220604_x_at | FTCD | 3,60E-02 | -0,204 |
| 206254_at | EGF | 3,61E-02 | -0,143 |
| 200679_x_at | HMGB1 | 3,62E-02 | -0,844 |
| 214268_s_at | MTMR4 | 3,62E-02 | 0,611 |
| 221471_at | SERINC3 | 3,62E-02 | 0,374 |
| 218457_s_at | DNMT3A | 3,65E-02 | -0,324 |
| 221337_s_at | ADAM29 | 3,67E-02 | 0,137 |
| 242302_at | PDS5B | 3,67E-02 | -0,086 |
| 229662_at | MSH3 | 3,70E-02 | -0,104 |
| 211210_x_at | SH2D1A | 3,71E-02 | -0,19 |
| 1553155_x_at | ATP6V0D2 | 3,73E-02 | 0,104 |
| 1559204_x_at | KRAS | 3,73E-02 | 0,305 |
| 223868_s_at | WWOX | 3,73E-02 | -0,292 |
| 209447_at | SYNE1 | 3,74E-02 | 0,479 |
| 216955_at | TAF1 | 3,74E-02 | 0,127 |
| 203057_s_at | PRDM2 | 3,75E-02 | 0,506 |
| 221325_at | KCNK13 | 3,75E-02 | -0,19 |
| 239132_at | NOS1 | 3,75E-02 | 0,134 |
| 1560080_at | DIAPH1 | 3,76E-02 | -0,087 |
| 207426_s_at | TNFSF4 | 3,76E-02 | -0,397 |
| 204446_s_at | ALOX5 | 3,77E-02 | -1,214 |
| 1405_i_at | CCL5 | 3,79E-02 | -1,04 |
| 213309_at | PLCL2 | 3,79E-02 | 0,681 |
| 209220_at | GPC3 | 3,81E-02 | -0,125 |
| 235746_s_at | PLA2R1 | 3,81E-02 | -0,128 |
| 206504_at | CYP24A1 | 3,82E-02 | 0,066 |
| 208894_at | HLA-DRA | 3,82E-02 | -1,119 |
| 219434_at | TREM1 | 3,83E-02 | -0,607 |
| 1552510_at | SLC34A3 | 3,84E-02 | 0,155 |
| 214222_at | DNAH7 | 3,85E-02 | -0,16 |
| 204068_at | STK3 | 3,86E-02 | 0,395 |
| 1552278_a_at | SLC46A1 | 3,87E-02 | 0,189 |
| 211542_x_at | RPS10 | 3,87E-02 | -0,198 |
| 226197_at | AR | 3,88E-02 | 0,246 |
| 204948_s_at | FST | 3,90E-02 | 0,349 |
| 208478_s_at | BAX | 3,90E-02 | -0,659 |
| 219743_at | HEY2 | 3,92E-02 | -0,604 |
| 217404_s_at | COL2A1 | 3,94E-02 | -0,127 |
| 1554942_a_at | KLHL14 | 3,95E-02 | -0,148 |
| 231000_at | ROR2 | 3,96E-02 | -0,183 |
| 242553_at | ABCC3 | 3,97E-02 | -0,117 |
| 1558378_a_at | AHNAK2 | 3,99E-02 | -0,23 |
| 203091_at | FUBP1 | 3,99E-02 | -0,282 |
| 204198_s_at | RUNX3 | 3,99E-02 | -0,958 |
| 207782_s_at | PSEN1 | 3,99E-02 | 0,46 |
| 204131_s_at | FOXO3 | 4,00E-02 | 0,686 |
| 212167_s_at | SMARCB1 | 4,00E-02 | -0,261 |
| 229004_at | ADAMTS15 | 4,00E-02 | -0,458 |
| 202878_s_at | CD93 | 4,01E-02 | -0,634 |
| 211000_s_at | IL6ST | 4,01E-02 | -0,55 |
| 214062_x_at | NFKBIB | 4,01E-02 | -0,173 |
| 216310_at | TAOK1 | 4,01E-02 | -0,163 |
| 239077_at | CSGALNACT2 | 4,01E-02 | -0,15 |
| 208591_s_at | PDE3B | 4,02E-02 | 0,181 |
| 219095_at | JMJD7-PLA2G4B | 4,02E-02 | -0,374 |
| 215568_x_at | LYPLA2 | 4,03E-02 | -0,191 |
| 232148_at | NSMAF | 4,03E-02 | 0,625 |
| 210186_s_at | FKBP1A | 4,04E-02 | -0,291 |
| 223916_s_at | BCOR | 4,05E-02 | -0,177 |
| 207691_x_at | ENTPD1 | 4,09E-02 | 1,068 |
| 227739_at | NDOR1 | 4,09E-02 | -0,192 |
| 239530_at | ADD2 | 4,10E-02 | -0,199 |
| 211154_at | THPO | 4,13E-02 | -0,077 |
| 200949_x_at | RPS20 | 4,14E-02 | -0,504 |
| 203266_s_at | MAP2K4 | 4,14E-02 | 0,395 |
| 202587_s_at | AK1 | 4,16E-02 | -0,496 |
| 200017_at | RPS27A | 4,17E-02 | -0,287 |
| 212977_at | ACKR3 | 4,17E-02 | 0,957 |
| 1552409_a_at | ODF4 | 4,19E-02 | 0,146 |
| 202659_at | PSMB10 | 4,19E-02 | -0,478 |
| 205936_s_at | HK3 | 4,19E-02 | -0,376 |
| 202411_at | IFI27 | 4,20E-02 | -1,401 |
| 204253_s_at | VDR | 4,21E-02 | -0,165 |
| 212816_s_at | CBS/LOC102724560 | 4,22E-02 | -0,927 |
| 204965_at | GC | 4,23E-02 | -0,079 |
| 205720_at | POMC | 4,26E-02 | -0,347 |
| 207129_at | CA5B | 4,26E-02 | 0,206 |
| 224991_at | CMIP | 4,31E-02 | -0,5 |
| 212276_at | LPIN1 | 4,34E-02 | -0,544 |
| 234524_at | PRDM15 | 4,34E-02 | -0,197 |
| 211396_at | FCGR2C | 4,37E-02 | -0,255 |
| 1555784_s_at | IRAK1 | 4,38E-02 | -0,328 |
| 214040_s_at | GSN | 4,39E-02 | -0,196 |
| 221301_at | VWA7 | 4,39E-02 | -0,144 |
| 206202_at | MEOX2 | 4,41E-02 | -0,072 |
| 220034_at | IRAK3 | 4,41E-02 | -0,293 |
| 1552811_at | WFIKKN1 | 4,43E-02 | -0,121 |
| 211113_s_at | ABCG1 | 4,43E-02 | -0,252 |
| 214219_x_at | MAP4K1 | 4,43E-02 | 0,393 |
| 240951_at | RORA | 4,43E-02 | -0,129 |
| 205262_at | KCNH2 | 4,44E-02 | -0,454 |
| 201094_at | RPS29 | 4,49E-02 | -0,122 |
| 205318_at | KIF5A | 4,49E-02 | 0,104 |
| 204162_at | NDC80 | 4,50E-02 | 0,743 |
| 208807_s_at | CHD3 | 4,52E-02 | -0,23 |
| 206074_s_at | HMGA1 | 4,53E-02 | -0,499 |
| 1562537_at | FCER1A | 4,54E-02 | 0,129 |
| 203105_s_at | DNM1L | 4,54E-02 | 0,312 |
| 201108_s_at | THBS1 | 4,62E-02 | -0,31 |
| 1555780_a_at | RHEB | 4,68E-02 | -0,534 |
| 208801_at | SRP72 | 4,68E-02 | 0,375 |
| 210730_s_at | NPY2R | 4,68E-02 | -0,139 |
| 213687_s_at | RPL35A | 4,68E-02 | -0,346 |
| 219497_s_at | BCL11A | 4,69E-02 | -0,914 |
| 203024_s_at | C5orf15 | 4,71E-02 | 0,489 |
| 234614_at | FLI1 | 4,72E-02 | -0,164 |
| 204124_at | SLC34A2 | 4,73E-02 | -0,091 |
| 1555018_at | OR2C3 | 4,75E-02 | -0,08 |
| 205841_at | JAK2 | 4,76E-02 | 0,331 |
| 200748_s_at | FTH1 | 4,79E-02 | -0,491 |
| 207056_s_at | SLC4A8 | 4,79E-02 | -0,11 |
| 219257_s_at | SPHK1 | 4,79E-02 | -0,498 |
| 1558331_at | SIRT2 | 4,80E-02 | -0,469 |
| 220764_at | PPP4R2 | 4,82E-02 | -0,214 |
| 224906_at | ANO6 | 4,82E-02 | 0,42 |
| 231169_at | TXLNA | 4,82E-02 | 0,407 |
| 1552663_a_at | ERC1 | 4,83E-02 | -0,115 |
| 1555102_at | FGF7 | 4,83E-02 | -0,079 |
| 243265_at | GAD2 | 4,83E-02 | -0,113 |
| 37950_at | PREP | 4,84E-02 | 0,347 |
| 227478_at | SETBP1 | 4,85E-02 | 0,655 |
| 238644_at | MYSM1 | 4,86E-02 | 0,288 |
| 209590_at | BMP7 | 4,91E-02 | 0,124 |
| 200854_at | NCOR1 | 4,95E-02 | 0,337 |
| 208383_s_at | PCK1 | 4,95E-02 | 0,106 |
| 208579_x_at | HIST1H2BK | 4,95E-02 | -0,931 |
| 208871_at | ATN1 | 4,95E-02 | -0,131 |
| 200793_s_at | ACO2 | 5,00E-02 | 0,451 |

Supplementary table S1: list of probe sets significantly differentially expressed in CR vs NR patients, filtered according to the GeneGo® “haematological disease” category.
